# Deep Reinforcement Learning-Based Energy-Efficient Resource Allocation and Scheduling in 6G-Enabled UAV-Assisted IoT Wireless Networks

**DOI:** 10.3390/s26175483

**Published:** 2026-08-29

**Authors:** Ali Nauman, Sung Won Kim

**Affiliations:** School of Computer Science and Engineering, College of Digital Convergence, Yeungnam University, Gyeongsan 38541, Republic of Korea

**Keywords:** 6G, Internet of Things (IoT), Deep Q-Network (DQN), Deep Reinforcement Learning (DRL), Energy Efficiency (EE), Unmanned Aerial Vehicles (UAVs)

## Abstract

Unmanned Aerial Vehicles (UAVs) have emerged as a flexible, cost-effective solution for connecting Internet of Things (IoT) devices where traditional infrastructure falls short. However, managing their limited energy alongside the diverse demands of densely deployed devices makes resource allocation a genuinely hard problem. This paper presents a Deep Reinforcement Learning (DRL) framework that jointly optimizes user scheduling, IoT device transmit power, bandwidth, and UAV movement in a 6G-enabled UAV-relay uplink network, using a deterministic large-scale air-to-ground path-loss channel model. The UAV acts as an aerial decode-and-forward relay between IoT devices and a Base Station (BS), with a Deep Q-Network (DQN) making decisions based on queue backlogs, channel conditions, UAV position, and remaining battery. The reward function balances Energy Efficiency (EE), queue stability, fairness, and battery longevity. We benchmark the DQN against six baselines; Round Robin (RR), Random Allocation (RA), the Single-to-Noise Ratio (Max-SNR), Proportional Fair (PF), a Lyapunov heuristic, and a GreedyEE scheme; across a range of device counts, traffic loads, battery budgets, and flight altitudes. Simulations consistently show that the DQN outperforms all baselines, including a RA baseline with equal access to UAV mobility; in EE, throughput, delay, and fairness, confirming that the gain stems from the learned joint control policy rather than from UAV mobility being available.

## 1. Introduction

The proliferation of Internet of Things (IoT) devices across industrial, agricultural, environmental, and urban monitoring domains is one of the defining technological trends of the 2020s. According to industry projections, the number of globally connected IoT endpoints is expected to exceed 39 billion by 2030, driving an unprecedented demand for low-latency, reliable, and energy-efficient wireless connectivity [1,2,3]. In many practical deployment scenarios; remote terrain monitoring, post-disaster search and rescue, smart agriculture, and offshore industrial sensing; IoT devices operate far from terrestrial Base Station (BS), and direct communication is impeded by large path loss, shadowing, or complete Line-of-Sight (LoS) obstruction due to terrain features [4,5].

Unmanned Aerial Vehicle (UAV)s offer an attractive solution to bridge this connectivity gap. Their ability to be rapidly deployed, repositioned, and recovered, combined with the inherently favorable air-to-ground channel conditions arising from their elevated altitude, makes UAVs compelling as aerial relays or mobile data collectors in IoT networks [6]. The emergence of 6G wireless technology further amplifies this potential: 6G promises to integrate Non-Terrestrial Network (NTN); including UAVs High-Altitude Platform Station(HAPS)s, and Low Earth Orbit (LEO) satellites; as native components of the communication infrastructure [7,8].

However, the effective deployment of UAVs as IoT relays is constrained by two fundamental challenges. First, UAVs operate on limited onboard battery capacity, which must sustain not only the energy consumed for data relay transmission but also the considerable propulsion and hovering energy required to remain airborne [9]. Second, the dynamic nature of the network; arising from time-varying wireless channels, stochastic IoT traffic arrivals, evolving queue states, and changing UAV position; makes it exceedingly difficult to derive a static optimal policy using conventional optimization methods [10].

The optimization of scheduling (which IoT device transmits), resource allocation (transmit power and bandwidth), and UAV trajectory in a battery-constrained decode-and-forward relay architecture constitutes a Mixed-Integer Non-Linear Programming (MINLP) problem with stochastic dynamics. Such problems are generally NP-hard even in deterministic settings [11,12], and the added stochasticity of wireless channels and traffic arrivals precludes the use of deterministic dynamic programming. This motivates the investigation of intelligent data-driven control, and specifically Deep Reinforcement Learning (DRL), as a means of learning adaptive policies that implicitly account for all these interdependencies [13,14].

### 1.1. Research Gap and Contributions

While a growing body of literature has investigated DRL for UAV-related optimization tasks (surveyed in Section 2), several research gaps remain.

1. Most prior work optimizes trajectory or resource allocation, but does not simultaneously determine IoT user scheduling, IoT device uplink transmit power levels, bandwidth fractions, and UAV movement direction in a unified DRL framework.
2. Battery sustainability as a first-class constraint is rarely modeled with explicit reward penalties and evaluated across varying battery budgets.
3. Fairness across heterogeneous IoT devices is often neglected or treated as a secondary metric rather than a reward component.
4. Comprehensive benchmarking against multiple heuristic baselines; including Lyapunov-inspired and Greedy Energy Efficiency (EE) schemes; under a range of network conditions is rarely provided.

This paper addresses all four gaps with the following contributions:

1. **Unified DRL-based control framework:** We propose a Deep Q-Network (DQN)-based controller that simultaneously determines IoT user scheduling, IoT device uplink transmit power levels, bandwidth fractions, and UAV movement direction at each time slot. Unlike decomposed approaches, the joint action representation allows the agent to learn cross-domain trade-offs.
2. **Battery-aware and fairness-enhanced reward design:** The reward function explicitly penalizes battery depletion events and incorporates a Jain fairness index term to prevent starvation of devices with persistently poor channel conditions.
3. **Scalable state representation:** The state vector is defined with a fixed maximum dimension regardless of the actual number of active IoT devices, enabling the trained policy to generalize across varying network sizes without retraining.
4. **Thorough multi-dimensional evaluation:** We benchmark the proposed DQN method against six alternative policies under four independent experiment axes: number of IoT devices, traffic arrival rate, UAV battery budget, and UAV altitude. This multi-axis evaluation provides a more complete picture of policy robustness than is typically reported.

### 1.2. Paper Organization

The remainder of this paper is organized as follows. Section 2 reviews related work. Section 3 describes the system model, including the network architecture, channel model, communication rates, queue dynamics, UAV mobility, and energy consumption model. Section 4 formally states the optimization problem and discusses the challenges motivating DRL. Section 5 presents the proposed DQN framework in detail, including Markov Decision Process (MDP) formulation, state and action space design, reward engineering, and training procedure. Section 6 provides the simulation setup. The results and discussion are presented in Section 7 and Section 8, provides future research directions, and Section 9 concludes the paper.

## 2. Related Work

### 2.1. UAV-Assisted Wireless Communication

The use of UAVs as communication infrastructure has attracted substantial research attention. In [6], the authors provided one of the earliest systematic overviews of UAV-enabled wireless communications, identifying trajectory optimization and energy management as central challenges. In [15], the authors studied joint trajectory and communication scheduling for UAV-enabled mobile relay and derived efficient successive convex approximation-based solutions. In [16], the authors presented a comprehensive tutorial on UAV-assisted communications, covering channel modeling, deployment optimization, and interference management. These works, while foundational, rely on deterministic convex optimization frameworks that require full knowledge of future channel states and traffic arrivals, limiting their applicability in dynamic and partially observable IoT scenarios.

### 2.2. EE in UAV Networks

EE in UAV communications has been studied from multiple angles. In [9], the authors analyzed the fundamental energy-throughput trade-off for UAV-enabled data collection and proposed optimal flight trajectories. In [17], the authors investigated joint power and trajectory optimization for UAV BS with EE as the primary objective. In [18], the authors examined energy-efficient resource allocation for multi-UAV networks using dynamic programming. A common limitation of these optimization approaches is the reliance on static or slowly-varying channel conditions and predetermined traffic models, which do not capture the fast-changing and stochastic nature of IoT sensor data.

### 2.3. DRL for UAV Control

The application of DRL to UAV communication has grown rapidly in recent years. In [19], the authors applied DQN to optimize UAV trajectory for data collection from multiple ground users. In [20], the authors proposed a multi-agent DRL approach for multi-UAV path planning and resource allocation. In [21], the authors developed a multi-agent actor-critic framework for joint computation offloading and UAV trajectory control in mobile edge computing scenarios. In [22], the authors provided a survey of DRL applications in communication networks, highlighting its suitability for stochastic resource management.

### 2.4. IoT Data Collection and Scheduling via UAVs

Several works have specifically targeted UAV-assisted IoT data collection. In [23], the authors proposed an Age-of-Information (AoI)-minimizing UAV scheduling policy for IoT sensor networks. In [24], the authors investigated energy-constrained UAV trajectory planning for IoT data gathering. In [25], the authors addressed multi-UAV cooperative data collection with fairness constraints. In [26], the authors applied DRL to minimize AoI in UAV-assisted vehicular IoT. Moreover, in [27], the authors proposed a CGAN-driven signal sample expansion to showcase that generative network-based data augmentation can effectively classify and mitigate jamming signals, making it useful for UAV-based communication and anti-interference performance. These works, however, either consider a simplified single-hop collection model or do not jointly optimize the full resource management tuple of scheduling, power, bandwidth, and trajectory.

### 2.5. Positioning of This Work

The present paper distinguishes itself from prior art in three key aspects. First, we consider a two-hop decode-and-forward relay architecture rather than a direct collection model, which introduces a bottleneck-limited rate and additional energy consumption at the UAV relay stage. Second, we propose a unified DQN framework that jointly and simultaneously optimizes all four control dimensions; scheduling, power of IoT devices, bandwidth, and trajectory; in a single agent, with a reward function that captures EE, queue stability, battery sustainability, and fairness. Third, we conduct a comprehensive evaluation under four independent variational axes and compare against six benchmark policies, providing a rigorous and balanced assessment of the proposed approach.

Moreover, it is worth clarifying the scope of the comparative evaluation in this paper: the six baselines (Round-Robin (RR), Random, Max Single-to-Noise Ratio (Max-SNR), Proportional Fair (PF), a Lyapunov heuristic, and GreedyEE) are classical and heuristic scheduling/resource-allocation policies representative of the UAV-IoT literature surveyed above, rather than alternative DRL architectures.

## 3. System Model

### 3.1. Network Architecture and Notation

**Figure 1:**
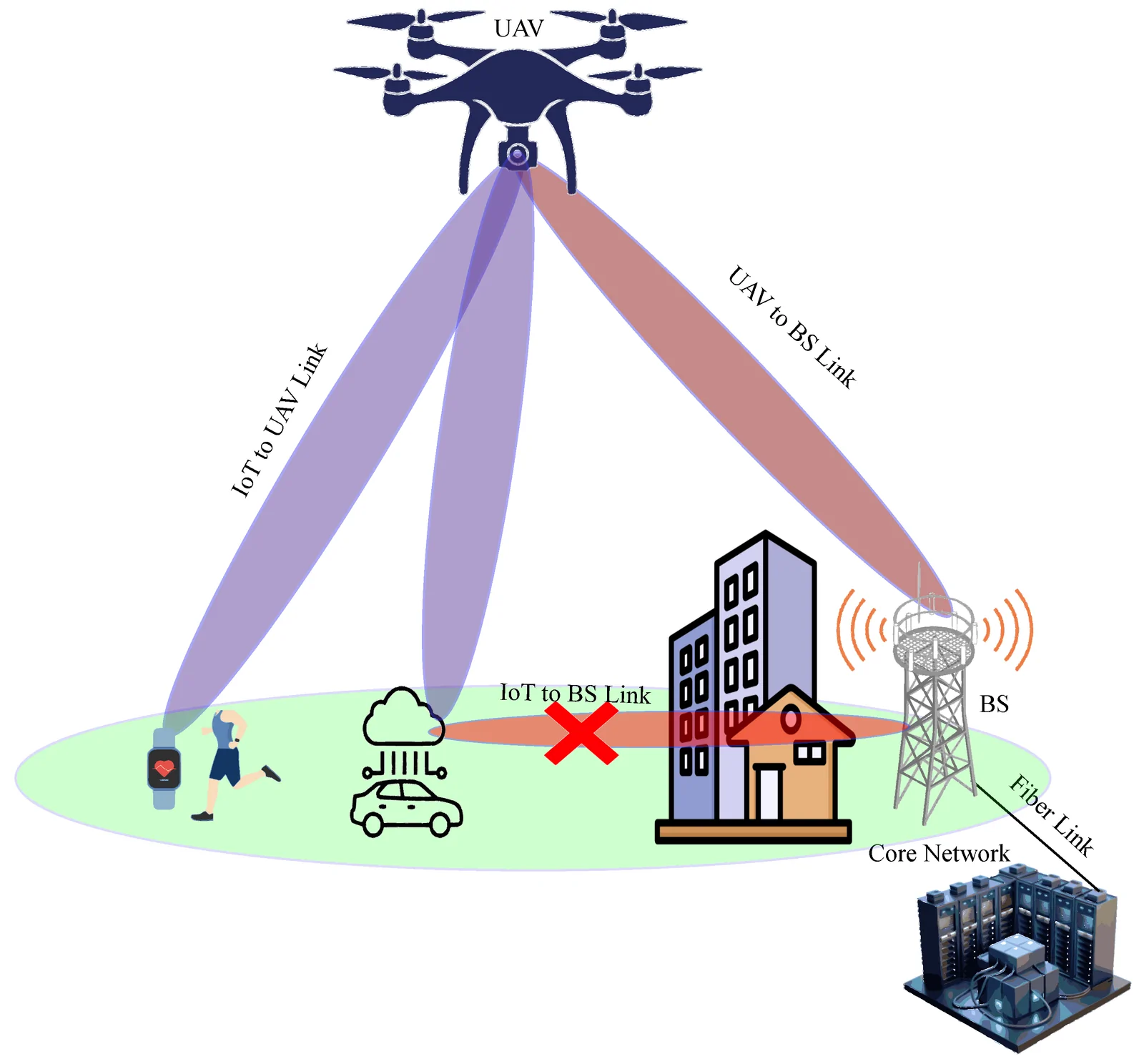
System model illustration.

We consider a UAV-assisted wireless IoT uplink network comprising *K* stationary ground IoT devices, one terrestrial BS, and a single UAV acting as an aerial relay, as illustrated in Figure 1. The direct communication link between the IoT devices and the BS is assumed to be blocked, severely attenuated, or otherwise unavailable due to path loss, terrain obstruction, or coverage constraints. Accordingly, all uplink data traffic is routed via the UAV relay in a two-hop architecture.

Let K={1,2,…,K} denote the set of IoT devices. The BS is located at a fixed two-dimensional position:

(1) $$w_{b}={\left[x_{b},\;y_{b}\right]}^{T}\in R^{2},$$

and the *k*-th IoT device is located at:

(2) $$w_{k}={\left[x_{k},\;y_{k}\right]}^{T},\;\;\;\;\forall k\in K.$$

The system operates over a finite horizon of *T* time slots indexed by T={1,2,…,T}, each of duration Δt seconds. The UAV flies at a fixed altitude *H* and its horizontal position at time slot *t* is denoted by:

(3) $$q\left(t\right)={\left[x_{u}\left(t\right),\;y_{u}\left(t\right)\right]}^{T}\in R^{2}.$$

The three-dimensional position is, therefore, (q(t),H). At most one IoT device is scheduled for uplink transmission per time slot. The scheduled device transmits data to the UAV (access link), which then decodes and forwards the data to the BS (backhaul link) using a decode-and-forward protocol.

### 3.2. Distance Model

The three-dimensional Euclidean distance between IoT device *k* and the UAV at slot *t* is:

(4) $$d_{k,u}\left(t\right)=\sqrt{∥q\left(t\right)-w_{k}{∥}^{2}+H^{2}},$$

and the distance between the UAV and the BS is:

(5) $$d_{u,b}\left(t\right)=\sqrt{∥q\left(t\right)-w_{b}{∥}^{2}+H^{2}}.$$

### 3.3. Channel Model

The elevated position of the UAV gives rise to dominant LoS propagation on both the IoT-to-UAV (access) link and the UAV-to-BS (backhaul) link. We adopt a deterministic large-scale path-loss channel model that is widely used for communication-oriented UAV studies [28,29,30].

#### 3.3.1. IoT-to-UAV Access Link

The channel power gain between IoT device *k* and the UAV at time slot *t* is:

(6) $$g_{k,u}\left(t\right)=\frac{\beta_{0}}{{\left(d_{k,u}\left(t\right)\right)}^{\alpha_{k,u}}},$$

where $\beta_{0}$ is the channel power gain at a reference distance of 1 m, and $\alpha_{k,u}$ is the path-loss exponent for the access link.

#### 3.3.2. UAV-to-BS Backhaul Link

Similarly, the channel power gain between the UAV and the BS is:

(7) $$g_{u,b}\left(t\right)=\frac{\beta_{0}}{{\left(d_{u,b}\left(t\right)\right)}^{\alpha_{u,b}}},$$

where $\alpha_{u,b}$ is the path-loss exponent of the backhaul link.

### 3.4. Scheduling and Resource Allocation

At each time slot *t*, the controller determines: (i) which IoT device is scheduled for uplink transmission, (ii) the transmit power assigned to the scheduled device, (iii) the bandwidth allocated to the scheduled transmission, and (iv) the UAV movement direction.

Let the binary scheduling variable be:

(8) $$a_{k}\left(t\right)=\left\{\begin{array}{cc} 1, & \mathrm{if}\;\mathrm{IoT}\;\mathrm{device}\;k\;\mathrm{is}\;\mathrm{scheduled}\;\mathrm{at}\;\mathrm{slot}\;t,\\ 0, & \mathrm{otherwise}, \end{array}\right.$$

subject to the single-user scheduling constraint:

(9) $$\sum_{k=1}^{K}a_{k}\left(t\right)\leq 1,\;\;\;\;\;\forall t\in T.$$

The uplink transmit power of device *k* at slot *t* is denoted by $p_{k}\left(t\right)$, subject to:

(10) $$0\leq p_{k}\left(t\right)\leq Pmax_{k},\;\;\;\;\;\forall k\in K,\;\forall t\in T.$$

The allocated access-link bandwidth to device *k* at slot *t* is $B_{k}\left(t\right)$, drawn from the shared IoT-access spectrum pool $B_{tot}$ (the UAV-BS backhaul link uses a separate dedicated band, and is therefore not subject to this pool):

(11) $$0\leq B_{k}\left(t\right)\leq B_{tot},\;\;\;\;\;\sum_{k=1}^{K}B_{k}\left(t\right)\leq B_{tot},\;\;\;\;\;\forall t.$$

Because at most one device is scheduled per slot, $B_{k}\left(t\right)=0$ for all unscheduled devices.

### 3.5. Achievable Transmission Rate and End-to-End Throughput

For the scheduled IoT device *k* at time slot *t*, the instantaneous achievable rate on the access link is given by Shannon’s capacity formula:

(12) $$R_{k,u}\left(t\right)=B_{k}\left(t\right)\log_{2}\;\left(1+\frac{p_{k}\left(t\right)g_{k,u}\left(t\right)}{N_{0}B_{k}\left(t\right)}\right),$$

where $N_{0}$ is the noise power spectral density. Similarly, the achievable rate on the backhaul link is:

(13) $$R_{u,b}\left(t\right)=B_{k}\left(t\right)\log_{2}\;\left(1+\frac{p_{u}\left(t\right)g_{u,b}\left(t\right)}{N_{0}B_{k}\left(t\right)}\right),$$

where $p_{u}\left(t\right)$ is the UAV relay transmit power. Under the decode-and-forward protocol, the end-to-end uplink rate for device *k* is bottlenecked by the weaker hop:

(14) $$R_{k}\left(t\right)=a_{k}\left(t\right)\min\;\left\{R_{k,u}\left(t\right),\;R_{u,b}\left(t\right)\right\}.$$

The access link (IoT-to-UAV) and the backhaul link (UAV-to-BS) are served by two physically separate, dedicated frequency bands: the access link uses bandwidth $B_{k}\left(t\right)$ drawn from the shared IoT-access spectrum pool $B_{tot}$ and allocated among IoT devices, while the backhaul link operates over a dedicated point-to-point UAV-BS band of fixed width $B_{bh}$ (e.g., a microwave/mmWave backhaul link) that is provisioned independently of $B_{tot}$ and does not compete with any IoT device access-link allocation; analogous to the dedicated backhaul/fiber connection from the BS to the core network shown in Figure 1. Equation (14) is accordingly rewritten with this dedicated parameter:

(15) $$R_{u,b}\left(t\right)=B_{bh}\log_{2}\;\left(1+\frac{p_{u}\left(t\right)g_{u,b}\left(t\right)}{N_{0}B_{bh}}\right).$$

In this study, we set $B_{bh}$ to be numerically equal to the tabulated access-link bandwidth values used for $B_{k}\left(t\right)$. Because the two hops occupy disjoint dedicated bands rather than time-sharing a single band, they may operate concurrently, and correctly captures the end-to-end throughput under this dedicated-backhaul-band architecture, with no additional time-sharing factor required. Relay-side buffering delay is treated as negligible relative to the slot duration, so no additional queuing delay is modeled at the UAV itself.

The total data successfully delivered from device *k* to the BS during slot *t* is:
  (16) $$D_{k}\left(t\right)=R_{k}\left(t\right)\cdot \Delta t.$$

### 3.6. Traffic Arrival and Queue Dynamics

Each IoT device maintains a local transmission queue. Let $Q_{k}\left(t\right)$ denote the queue backlog (in bits) of device *k* at the beginning of slot *t*, and $A_{k}\left(t\right)$ the amount of data arriving at device *k* during slot *t*. The queue evolves as:

(17) $$Q_{k}\left(t+1\right)=\min\;\left\{\max\;\left\{Q_{k}\left(t\right)-D_{k}\left(t\right),\;0\right\}+A_{k}\left(t\right),\;Qmax_{k}\right\},$$

where $Qmax_{k}$ is the maximum buffer size of device *k*.

Thearrival process $A_{k}\left(t\right)$ is modeled as independent and identically distributed Poisson random variables with mean $\lambda_{k}$ bits per slot:

(18) $$A_{k}\left(t\right)∼\mathrm{Poisson}\left(\lambda_{k}\right),\;\;\;\;\;EE\left[A_{k}\left(t\right)\right]=\lambda_{k}.$$

This models bursty IoT sensor traffic (e.g., periodic environmental measurements with random packet sizes) in a tractable manner.

### 3.7. UAV Mobility Model

The UAV moves horizontally at a fixed altitude, *H*, within a bounded service area, $A=\left[0,L_{x}\right]\times \left[0,L_{y}\right]$. The mobility constraint limits the maximum displacement per slot:

(19) ∥q(t+1)−q(t)∥≤Vmax·Δt,     ∀t∈T,

where Vmax is the maximum horizontal speed. For the DRL implementation, the continuous movement space is discretized into five actions: M={hover,north,south,east,west}, corresponding to a fixed step size of $\delta_{xy}$ meters per slot. The service area boundary is enforced by clipping the UAV position:

(20) q(t)∈A,     ∀t∈T.

#### 3.7.1. IoT Device Transmission Energy

When device *k* is scheduled at slot *t*, its uplink transmission energy is:

(21) $$E_{k}^{\mathrm{tx}}\left(t\right)=a_{k}\left(t\right)\;p_{k}\left(t\right)\;\Delta t.$$

#### 3.7.2. UAV Relay Transmission Energy

The UAV forwards decoded data to the BS using a fixed relay transmit power $p_{u}$, consuming:

(22) $$E_{u}^{\mathrm{relay}}\left(t\right)=p_{u}\;\Delta t.$$

#### 3.7.3. UAV Propulsion and Hovering Energy

UAV propulsion energy accounts for both the static hovering cost and the additional cost incurred when the UAV moves. We adopt the simplified discrete-time model:

(23) $$E_{u}^{\mathrm{mob}}\left(t\right)=E_{\mathrm{hov}}+\kappa \cdot ∥q\left(t+1\right)-q\left(t\right)∥,$$

where $E_{\mathrm{hov}}$ is the hovering energy per slot (in joules), and κ is a propulsion energy coefficient (J/m).

#### 3.7.4. Circuit Energy

The communication circuitry of the UAV and scheduled IoT device consumes a constant circuit power $P_{c}$ per slot:

(24) $$E^{\mathrm{cir}}\left(t\right)=P_{c}\;\Delta t.$$

#### 3.7.5. Total System Energy Consumption

Combining all components, the total system energy consumed in slot *t* is:

(25) $$E_{\mathrm{tot}}\left(t\right)=\sum_{k=1}^{K}E_{k}^{\mathrm{tx}}\left(t\right)+E_{u}^{\mathrm{relay}}\left(t\right)+E_{u}^{\mathrm{mob}}\left(t\right)+E^{\mathrm{cir}}\left(t\right).$$

### 3.8. UAV Battery Model

The UAV carries a finite onboard battery with initial energy $E_{u}^{\max}$. The residual battery energy evolves as:

(26) $$Erem_{u}\left(t+1\right)=Erem_{u}\left(t\right)-E_{u}^{\mathrm{relay}}\left(t\right)-E_{u}^{\mathrm{mob}}\left(t\right)-E^{\mathrm{cir}}\left(t\right),$$

with the constraint:

(27) $$Erem_{u}\left(t\right)\geq 0,\;\;\;\;\;\forall t\in T,$$

and initial condition $Erem_{u}\left(1\right)=E_{u}^{\max}$. Battery depletion ($Erem_{u}\left(t\right)=0$) terminates the operation episode in our simulation, modeling the physical constraint that a UAV must return to base for recharging.

### 3.9. EE Metric

The primary performance metric is the long-term average EE, defined as the ratio of the total data delivered to the BS over the total energy consumed:

(28) $$\eta_{\mathrm{avg}}=\frac{\sum_{t=1}^{T}\sum_{k=1}^{K}D_{k}\left(t\right)}{\sum_{t=1}^{T}E_{\mathrm{tot}}\left(t\right)},$$

measured in bits/joule (bits/J). This metric captures the fundamental trade-off between data delivery and energy expenditure over the full mission horizon.

### 3.10. Queue Stability and Delay Considerations

In addition to EE, the system must maintain acceptable end-to-end delay. By Little’s law, the average delay experienced by packets of device *k* is proportional to its mean queue length ${\bar{Q}}_{k}$. Accordingly, a queue is said to be strongly stable if:

(29) $$\underset{T\to \infty }{\mathrm{lim sup}}\frac{1}{T}\sum_{t=1}^{T}EE\left[Q_{k}\left(t\right)\right]<\infty ,\;\;\;\;\;\forall k\in K.$$

The control policy should therefore prevent unbounded queue accumulation while maximizing EE.

### 3.11. Fairness Metric

To assess equitable service distribution among IoT devices, we use Jain’s fairness index:

(30) $$J=\frac{{\left(\sum_{k=1}^{K}{\bar{D}}_{k}\right)}^{2}}{K\sum_{k=1}^{K}{\bar{D}}_{k}^{2}},$$

where ${\bar{D}}_{k}=\sum_{t=1}^{T}D_{k}\left(t\right)$ is the cumulative data served for device *k* over the mission horizon. The index satisfies J∈(0,1], with J=1 indicating perfect fairness.

## 4. Problem Formulation

### 4.1. Design Objective

Let the total data delivered to the BS at slot *t* be $D_{\mathrm{sum}}\left(t\right)=\sum_{k=1}^{K}D_{k}\left(t\right)$, and let the corresponding total energy consumption be $E_{\mathrm{tot}}\left(t\right)$ as defined in (25). The goal is to find a control policy Π={u(1),u(2),…,u(T)} over the slot-wise action $u\left(t\right)=\left(\left\{a_{k}\left(t\right)\right\},\left\{p_{k}\left(t\right)\right\},\left\{B_{k}\left(t\right)\right\},bq\left(t+1\right)\right)$ that maximizes the long-term average EE (28).

### 4.2. Optimization Problem

The joint energy-efficient resource allocation problem is formally stated as:

(31a) $$\begin{array}{cc} P1:\;\max_{\Pi }\; & \eta_{\mathrm{avg}}=\frac{\sum_{t=1}^{T}D_{\mathrm{sum}}\left(t\right)}{\sum_{t=1}^{T}E_{\mathrm{tot}}\left(t\right)} \end{array}$$

(31b) $$\begin{array}{cc} s.t.\;\; & \sum_{k=1}^{K}a_{k}\left(t\right)\leq 1,\;\;\;\;\;\forall t, \end{array}$$

(31c) $$\begin{array}{cc} & a_{k}\left(t\right)\in \left\{0,1\right\},\;\;\;\;\;\forall k,t, \end{array}$$

(31d) $$\begin{array}{cc} & 0\leq p_{k}\left(t\right)\leq Pmax_{k},\;\;\;\;\;\forall k,t, \end{array}$$

(31e) $$\begin{array}{cc} & 0\leq B_{k}\left(t\right)\leq B_{tot},\;\;\;\;\;\forall k,t, \end{array}$$

(31f) $$\begin{array}{cc} & \sum_{k=1}^{K}B_{k}\left(t\right)\leq B_{tot},\;\;\;\;\;\forall t, \end{array}$$

(31g) $$\begin{array}{cc} & Q_{k}\left(t+1\right)=\min\;\left\{\max\;\left\{Q_{k}\left(t\right)-D_{k}\left(t\right),0\right\}+A_{k}\left(t\right),Qmax_{k}\right\},\forall k,t, \end{array}$$

(31h) $$\begin{array}{cc} & 0\leq Q_{k}\left(t\right)\leq Qmax_{k},\;\;\;\;\;\forall k,t, \end{array}$$

(31i) $$\begin{array}{cc} & ∥q(t+1)-q(t)∥\leq Vmax\Delta t,\;\;\;\;\;\forall t, \end{array}$$

(31j) $$\begin{array}{cc} & q(t)\in A,\;\;\;\;\;\forall t, \end{array}$$

(31k) $$\begin{array}{cc} & Erem_{u}\left(t+1\right)=Erem_{u}\left(t\right)-E_{u}^{\mathrm{relay}}\left(t\right)-E_{u}^{\mathrm{mob}}\left(t\right)-E_{u}^{\mathrm{cir}}\left(t\right),\forall t, \end{array}$$

(31l) $$\begin{array}{cc} & Erem_{u}\left(t\right)\geq 0,\;\;\;\;\;\forall t. \end{array}$$

Constraint (31b) enforces single-user scheduling per slot, while (31c) defines the binary scheduling nature. Constraints (31d)–(31f) govern transmit power and bandwidth. Constraints (31g)–(31h) define queue evolution and buffer limits. Constraints (31i)–(31j) enforce UAV mobility feasibility. Finally, (31k)–(31l) capture UAV battery dynamics.

### 4.3. Challenges of Solving **P1**

Problem **P1** is fundamentally challenging due to four interrelated properties:

#### 4.3.1. Non-Convex Fractional Objective

The objective in (31a) is a ratio of two coupled sums, both of which depend nonlinearly on the continuous variables $p_{k}\left(t\right)$, $B_{k}\left(t\right)$, and q(t). Even without the integer constraints, such fractional objectives are generally non-convex. Dinkelbach’s method can transform the fractional objective into a parameterized concave-minus-convex form, but the coupling across time slots and the presence of binary variables preclude direct application.

#### 4.3.2. MINLP

The scheduling variables $a_{k}\left(t\right)\in \left\{0,1\right\}$ make **P1** a MINLP problem. MINLP is generally NP-hard, and the combinatorial explosion of the scheduling space ($2^{K}$ per slot) makes exact branch-and-bound methods computationally intractable for real-time control with K≥6.

#### 4.3.3. Temporal Coupling

The control decisions are strongly coupled across time through three state variables: queue backlogs (31g), UAV battery (31k), and UAV position (31i). An action taken at slot *t* alters the system state and thus the reward achievable at all future slots. This temporal dependency renders **P1** a multi-period stochastic program that cannot be decomposed into independent single-slot subproblems without approximation.

#### 4.3.4. Stochastic Traffic and Channels

The Poisson traffic arrivals $A_{k}\left(t\right)$ and the time-varying channel gains induced by UAV movement introduce stochasticity that makes deterministic dynamic programming intractable. Stochastic dynamic programming via Bellman’s equation requires enumeration of the continuous state space, which is computationally infeasible for the considered network dimensions.

### 4.4. Motivation for DRL

The above challenges—non-convexity, mixed-integer structure, temporal coupling, and stochastic dynamics—collectively motivate the use of DRL as a solution methodology. DRL avoids the need for an explicit model of state transitions and channel statistics; instead, the agent learns an effective control policy purely from observed transitions. By approximating the optimal action-value function with a deep neural network, the agent can generalize across the continuous and high-dimensional state space. Critically, the temporal coupling across queue, battery, and trajectory states is naturally handled through the discounted cumulative reward formulation, enabling the agent to learn long-horizon trade-offs that myopic heuristics cannot capture. The discounted per-slot reward is adopted here as a tractable training signal; the resulting policy quality with respect to the mission-level objective $\eta_{avg}$ is assessed directly in results using the exact metric defined, independent of the surrogate reward used during training.

## 5. Proposed DRL Framework

### 5.1. MDP Formulation

We model the UAV-assisted IoT uplink control problem as an MDP characterized by the tuple M=(S,A,P,R,γ), where S is the state space, A is the action space, P is the state transition probability, R is the reward function, and γ∈(0,1] is the discount factor. The objective is to learn a policy, π:S→A, that maximizes the expected discounted cumulative reward:

(32) $$\max_{\pi }\;EE_{\pi }\;\left[\sum_{t=1}^{T}\gamma^{t-1}r\left(t\right)\right].$$

### 5.2. State Space Design

The state at time slot *t* is defined as:

(33) $$bs\left(t\right)=\left[Q\left(t\right),\;G_{u}\left(t\right),\;g_{u,b}\left(t\right),\;q\left(t\right),\;Erem_{u}\left(t\right)\right],$$

where:

- $Q\left(t\right)=[Q_{1}\left(t\right),\ldots ,Q_{K}\left(t\right)]$: IoT queue backlogs;
- $G_{u}\left(t\right)=\left[g_{1,u}\left(t\right),\ldots ,g_{K,u}\left(t\right)\right]$: IoT-to-UAV channel gains;
- $g_{u,b}\left(t\right)$: UAV-to-BS channel gain;
- $q\left(t\right)={\left[x_{u}\left(t\right),y_{u}\left(t\right)\right]}^{T}$: UAV horizontal position;
- $Erem_{u}\left(t\right)$: remaining UAV battery.

To ensure a fixed state dimension that accommodates varying numbers of active IoT devices without retraining, the state vector is defined over $K_{\max}$ slots, yielding a dimension of $N_{S}=2K_{\max}+4$. All state variables are normalized before being fed into the neural network:

(34) $${\hat{Q}}_{k}\left(t\right)=\frac{Q_{k}\left(t\right)}{Qmax_{k}},\;\;\;\;\;{\hat{E}}_{u}\left(t\right)=\frac{Erem_{u}\left(t\right)}{E_{u}^{\max}},\;\;\;\;\;{\hat{g}}_{k,u}\left(t\right)=\frac{g_{k,u}\left(t\right)}{\beta_{0}},\;\;\;\;\;{\hat{x}}_{u}\left(t\right)=\frac{x_{u}\left(t\right)}{L_{x}},\;\;\;\;\;{\hat{y}}_{u}\left(t\right)=\frac{y_{u}\left(t\right)}{L_{y}}.$$

An additional binary active-device ratio $K_{\mathrm{active}}/K_{\max}$ is appended to the state to allow the agent to adapt its behavior to the actual number of devices present.

### 5.3. Action Space Design

The action at time slot *t* jointly determines the scheduled IoT device, the uplink transmit power level, the bandwidth fraction, and the UAV movement direction:

(35) $$a\left(t\right)=(k^{*}\left(t\right),\;p^{\mathrm{idx}}\left(t\right),\;B^{\mathrm{idx}}\left(t\right),\;m\left(t\right)),$$

where:

- $k^{*}\left(t\right)\in K$: selected IoT device;
- $p^{\mathrm{idx}}\left(t\right)\in P=\left\{P^{\left(1\right)},P^{\left(2\right)},P^{\left(3\right)},P^{\left(4\right)}\right\}$: transmit power level;
- $B^{\mathrm{idx}}\left(t\right)\in B=\left\{B^{\left(1\right)},B^{\left(2\right)},B^{\left(3\right)},B^{\left(4\right)}\right\}$: bandwidth fraction;
- m(t)∈M={hover,N,S,E,W}: UAV movement direction.

The total number of discrete actions is:

(36) $$\left|A\right|=K_{\max}\times N_{P}\times N_{B}\times N_{M},$$

where $N_{P}=\left|P\right|$, $N_{B}=\left|B\right|$, and $N_{M}=\left|M\right|$. In our implementation, $N_{P}=N_{B}=4$ and $N_{M}=5$, giving |A|=10×4×4×5=800 actions for $K_{\max}=10$.

The discrete power levels and bandwidth fractions used in the simulation are:

(37) $$P=\left\{0.05,0.10,0.20,0.35\right\}\;W,\;\;\;\;\;B=\left\{0.20,0.35,0.50,0.70\right\}\times B_{\mathrm{tot}}.$$

This discretization provides a meaningful coverage of the feasible resource space while keeping the action space manageable for DQN convergence. It is noted that only the IoT device’s transmit power is included in the learned action space.

### 5.4. Reward Function Design

The reward function must translate the multi-objective optimization goal—EE, queue stability, battery sustainability, fairness—into a scalar signal that guides the DQN towards the desired behavior. We define the per-slot reward as:

(38) $$r\left(t\right)=\lambda_{1}\eta \left(t\right)-\lambda_{2}\sum_{k=1}^{K}Q_{k}\left(t\right)-\lambda_{3}\Phi \;\left(Erem_{u}\left(t\right)\right)+\lambda_{4}F\left(t\right)-\lambda_{5}I\left(t\right),$$

where:

- $\eta \left(t\right)=D_{\mathrm{sum}}\left(t\right)/E_{\mathrm{tot}}\left(t\right)$ is the instantaneous EE (bits/J);
- $\lambda_{1},\ldots ,\lambda_{5}\geq 0$ are design hyperparameters;
- $\Phi (Erem_{u}\left(t\right))$ is a battery depletion penalty;
- F(t) is a fairness term;
- I(t) is an invalid-action penalty.

We emphasize that the per-slot reward is used exclusively as a training-time shaping signal that guides the DQN gradient updates; it is not itself the quantity used to evaluate policy quality. In particular, its EE component is a per-slot ratio, and maximizing $\sum_{t}\gamma^{t-1}r\left(t\right)$ is not, in general, equivalent to maximizing the mission-level ratio-of-sums objective. The DQN is, therefore, trained with a convenient per-slot surrogate but is scored, exactly like every baseline, on the true mission-level objective $\eta_{avg}$, consistent with standard reward-shaping practice in Reinforcement Learning (RL).

#### 5.4.1. Battery Penalty

A soft penalty proportional to battery under-threshold violation is used:

(39) $$\Phi \left(Erem_{u}\left(t\right)\right)=\max\;\left\{0,\;E_{\mathrm{th}}-Erem_{u}\left(t\right)\right\},$$

where $E_{\mathrm{th}}$ is a safety threshold. When the battery falls below $E_{\mathrm{th}}$, the penalty grows linearly, discouraging energy-wasteful actions near the end of the mission. A hard terminal penalty of −5 is applied when $Erem_{u}\left(t\right)\leq 0$, terminating the episode.

#### 5.4.2. Fairness Term

To promote equitable service across IoT devices, the per-slot fairness component uses a running Jain’s index:

(40) $$F\left(t\right)=\frac{{\left(\sum_{k=1}^{K}{\bar{D}}_{k}\left(t\right)\right)}^{2}}{K\sum_{k=1}^{K}{\bar{D}}_{k}{\left(t\right)}^{2}+\epsilon },$$

where ${\bar{D}}_{k}\left(t\right)=\sum_{\tau =1}^{t}D_{k}\left(\tau \right)+10^{-9}$ is the cumulative data served for device *k* up to slot *t*, and ϵ>0 prevents division by zero. It is noted that (40) is a ratio of a squared sum to *K* times a sum of squares, it is invariant to uniform scaling of the cumulative throughputs and if scaled by the same constant, it is unchanged. Consequently, F(t) remains bounded in (0, 1] throughout the training horizon and does not introduce reward-scale drift; what does change as *t* grows is only its sensitivity to short-term imbalances, since early-slot outcomes are increasingly diluted by accumulated history.

#### 5.4.3. Invalid-Action Penalty

If the action schedules an inactive or empty-queue device, an additional penalty is applied:

(41) $$I\left(t\right)=1\;\left[\mathrm{action}\;\mathrm{is}\;\mathrm{infeasible}\;\mathrm{at}\;\mathrm{slot}\;t\right].$$

This discourages the agent from wasting slot opportunities on devices that have no data to transmit. It is noted that a soft invalid-action penalty is used rather than hard action masking during training because it keeps the network’s fixed dimensional output head unchanged across the multi-environment training procedure with varying $K_{\mathrm{active}}$, avoiding the need to reconstruct a per-episode action mask for each sampled $K_{\mathrm{active}}$.

#### 5.4.4. Reward Coefficient Selection

The reward weighting coefficients in (38) are set as $\lambda_{1}=1.0$, $\lambda_{2}=4\times 10^{-6}$, $\lambda_{3}=0.01$, $\lambda_{4}=0.10$, and $\lambda_{5}=2.0$. These values are chosen so that the queue penalty term has a magnitude comparable to the EE term at typical queue occupancies, while the fairness and battery terms serve as regularizers. The reward weights were selected via a coarse offline grid search during development to balance the relative magnitudes of the EE, queue-penalty, battery-penalty, and fairness terms at typical operating points.

### 5.5. DQN Architecture

Because the state space is high-dimensional and continuous, tabular Q-learning is infeasible. Instead, we use a deep neural network parameterized by θ to approximate the action-value function:

(42) $$Q\left(s,a;\;\theta \right)\approx Q^{*}\left(s,a\right),$$

where $Q^{*}\left(s,a\right)$ is the optimal action-value function satisfying the Bellman optimality equation:

(43) $$Q^{*}\left(s,a\right)=EE\;\left[r\left(t\right)+\gamma \max_{a^{\prime }\in A}Q^{*}\left(s^{\prime },a^{\prime }\right)\;|\;s\left(t\right)=ss,a\left(t\right)=a\right].$$

The DQN architecture consists of:

- **Input layer:** receives the normalized state vector $\hat{s}\left(t\right)\in R^{N_{S}}$;
- **Hidden layer 1:** ReLU activation, fully connected, 128 neurons;
- **Hidden layer 2:** ReLU activation, fully connected, 128 neurons;
- **Output layer:** |A| neurons (one Q-value per action), fully connected.

The output vector is $Q\left(\hat{s}\left(t\right);\;\theta \right)\in R^{\left|A\right|}$, and the greedy action is $\pi \left(s\right)=\arg\max_{a}Q\left(s,a;\;\theta \right)$.

### 5.6. Experience Replay

To improve sample efficiency and break temporal correlations between consecutive transitions, an experience replay buffer D with capacity *C* is maintained. At each step, the transition tuple (s(t),a(t),r(t),s(t+1),done) is stored in D. During training, mini-batches of size $N_{\mathrm{batch}}$ are sampled uniformly from D to update the network parameters. This mechanism provides two benefits: (i) each transition may be used in multiple gradient updates, improving data efficiency; and (ii) the uniform sampling decorrelates the training data, reducing the variance of gradient estimates.

### 5.7. Target Network

Directly using the online network Q(s,a; θ) to compute both the current Q-values and the bootstrap targets introduces harmful feedback loops that can destabilize training. To mitigate this, a target network $Q(s,a;\;\theta^{-})$ with a periodically synchronized copy of the parameters is used. The target Q-value for a transition is:

(44) $$y\left(t\right)=\left\{\begin{array}{cc} r(t), & \mathrm{if}\;\mathrm{terminal},\\ r\left(t\right)+\gamma \max_{a^{\prime }}Q\left(s\left(t+1\right),a^{\prime };\;\theta^{-}\right), & \mathrm{otherwise}. \end{array}\right.$$

The target parameters $\theta^{-}$ are synchronized with the online parameters θ every $N_{\mathrm{target}}$ gradient steps.

### 5.8. Loss Function and Optimization

The DQN is trained by minimizing the mean squared temporal difference error over sampled mini-batches:

(45) $$L\left(\theta \right)=EE_{(s,a,r,s^{\prime })∼D}\;\left[{\left(y-Q(s,a;\;\theta )\right)}^{2}\right].$$

Gradient descent is performed using the Adam optimizer with learning rate α:

(46) $$\theta \leftarrow \theta -\alpha \nabla_{\theta }L\left(\theta \right).$$

### 5.9. Exploration-Exploitation Strategy

The agent follows an ϵ-greedy policy during training:

(47) $$a\left(t\right)=\left\{\begin{array}{cc} \mathrm{random}\;\mathrm{action}\;\mathrm{from}\;A, & \mathrm{with}\;\mathrm{probability}\;\epsilon ,\\ \arg\max_{a\in A}Q\left(s\left(t\right),a;\;\theta \right), & \mathrm{with}\;\mathrm{probability}\;1-\epsilon . \end{array}\right.$$

The exploration rate is initialized at $\epsilon_{\mathrm{start}}=1.0$ and decayed multiplicatively after each episode:

(48) $$\epsilon \leftarrow \max\left(\epsilon_{\min},\;\rho \cdot \epsilon \right),$$

where ρ=0.992 is the decay factor, and $\epsilon_{\min}=0.05$. This schedule encourages broad exploration early in training and progressively shifts to exploitation as the Q-function estimate matures.

### 5.10. Training Procedure

The complete DQN training procedure is summarized in Algorithm 1, which proceeds through seven stages at each time step:

1. State extraction and normalization, producing the fixed-dimension normalized state vector.
2. ϵ-greedy action selection over the joint action space.
3. Environment execution, in which the selected scheduling, power, bandwidth, and movement decisions are applied, and the reward is computed.
4. Storage of the resulting transition in the experience replay buffer.
5. Mini-batch sampling from the replay buffer once sufficient transitions are available.
6. Target-value computation using the target network and a gradient step on the online network via Adam.
7. Periodic synchronization of the target network parameters with the online network every target steps.

**Algorithm 1** DQN-based energy-efficient resource allocation and scheduling.**Initialize:** replay memory D with capacity *C*; online DQN with parameters θ; target DQN with $\theta^{-}\leftarrow \theta$; exploration rate $\epsilon \leftarrow \epsilon_{\mathrm{start}}$; step counter τ←0**for** episode $=1,2,\ldots ,N_{\mathrm{ep}}$ **do**      Sample $K_{\mathrm{active}}$ uniformly from {4,6,8,10}      Reset environment, obtain initial state s(1)      **for** t=1,2,…,T **do**            With probability ϵ: select random action a(t); else $a\left(t\right)=\arg\max_{a}Q\left(s\left(t\right),a;\theta \right)$            Execute a(t); observe reward r(t) and next state s(t+1); observe done flag            Store (s(t),a(t),r(t),s(t+1),done) in D            τ←τ+1            **if** $\left|D|\geq \max(N_{\mathrm{batch}},N_{\mathrm{start}}\right)$ **then**                 Sample mini-batch ${\left\{\left(s_{i},a_{i},r_{i},s_{i}^{\prime },d_{i}\right)\right\}}_{i=1}^{N_{\mathrm{batch}}}$ from D                 Compute targets $y_{i}$ using (44) with $\theta^{-}$                 Minimize $L\left(\theta \right)=\frac{1}{N_{\mathrm{batch}}}\sum_{i}{\left(y_{i}-Q\left(s_{i},a_{i};\theta \right)\right)}^{2}$ via Adam                 **if** $\tau \;\mathrm{mod}\;N_{\mathrm{target}}=0$ **then**                       $\theta^{-}\leftarrow \theta$                 **end if**            **end if**            **if** done **then**                 **break**            **end if**      **end for**      $\epsilon \leftarrow \max(\epsilon_{\min},\;\rho \cdot \epsilon )$**end for****return** trained online DQN with parameters $\theta^{*}$

### 5.11. Multi-Environment Training for Generalization

A key design choice in the training procedure is the random selection of $K_{\mathrm{active}}$ at the start of each episode from the set {4,6,8,10}. This multi-environment training strategy exposes the agent to diverse network configurations and encourages the learned policy to generalize across different numbers of active IoT devices without retraining. The fixed-size state vector and the active-device ratio appended to the state provide the agent with the necessary context to distinguish the current operating condition.

### 5.12. Online Deployment

After training, the agent operates in a purely greedy fashion: at each slot *t*, the normalized state $\hat{s}\left(t\right)$ is passed through the trained DQN, and the action with the highest predicted Q-value is selected:

(49) $$a^{*}\left(t\right)=\arg\max_{a\in A}Q\left(\hat{s}\left(t\right),a;\;\theta^{*}\right).$$

The forward pass through the two-hidden-layer DQN requires only $O(N_{S}\cdot h_{1}+h_{1}\cdot h_{2}+h_{2}\cdot |A|)$ multiply-accumulate operations per slot; far less computationally demanding than solving an MINLP at every slot; making the approach suitable for real-time deployment on embedded UAV hardware.

### 5.13. Complexity Analysis

Let $N_{S}$ denote the state dimension, $h_{1}$ and $h_{2}$ the widths of the two hidden layers, and $N_{A}=\left|A\right|$ the number of actions. The parameter count of the DQN is $\Theta =N_{S}h_{1}+h_{1}h_{2}+h_{2}N_{A}+h_{1}+h_{2}+N_{A}$. For $N_{S}=24$, $h_{1}=h_{2}=128$, and $N_{A}=800$, this amounts to approximately 119K parameters, which is modest by Deep Learning (DL) standards. The training complexity per gradient step is $O(N_{\mathrm{batch}}\cdot \Theta )$, and the total training budget is $N_{\mathrm{ep}}\times T$ environment steps. Online inference requires only a single forward pass of $O(\Theta /N_{A})$ operations per time slot.

## 6. Simulation Setup

### 6.1. Network Parameters

Simulations are conducted in MATLAB 2026a using the DL Toolbox Version 26.1 The network parameters are summarized in Table 1. Also, it is noted that UAV hovering energy/slot is expressed as a normalized per-slot energy unit calibrated for the simulated micro-scale UAV/IoT testbed, rather than as an absolute physical hover-power measurement.

#### Baseline Definitions

To ensure reproducibility, the exact decision rule of each baseline is defined as follows:

1. RR: Devices are scheduled in a fixed cyclic order regardless of queue or channel state, with transmit power and bandwidth fixed at mid-range values and the UAV hovering at a fixed position.
2. Random Allocation (RA): At each slot, one active device, power level, bandwidth fraction, and UAV movement direction are each selected uniformly at random from their respective sets.
3. Max-SNR: the device with the highest instantaneous access-link channel gain is scheduled using the maximum power level and bandwidth fraction; the UAV hovers.
4. PF: The device maximizing the ratio of its instantaneous achievable rate to its exponentially averaged historical rate is scheduled, with power and bandwidth fixed at mid-range values.
5. Lyapunov Heuristic: A drift-plus-penalty virtual-queue scheduler with fixed control parameter selects, at each slot, the device maximizing the weighted product of its queue backlog and achievable rate, with power and bandwidth chosen to maximize this weighted metric subject to the constraint set; the UAV hovers.
6. GreedyEE: At each slot, the device/power/bandwidth combination maximizing the instantaneous per-slot EE is selected greedily, with no consideration of queue backlog or fairness; the UAV hovers. This purely myopic, EE-only objective is why GreedyEE exhibits higher delay and lower fairness than PF, RR, and the DQN.

It is noted that the proposed DQN jointly controls scheduling, transmit power, bandwidth, and the UAV movement within a single learned policy, consistent with the central contribution of a unified joint-control framework. The hovering baselines are representative of the classical, decomposed resource-allocation literature surveyed in Section 2, against which this unified framework is positioned, and are therefore intentionally evaluated with a fixed UAV position rather than as an ablation of the DRL algorithm alone. However, the RA baseline already has access to the identical action space as the DQN.

### 6.2. Evaluation Protocol

After training, each method is evaluated over 20 independent evaluation episodes, each consisting of 80 time slots, with fresh random IoT device positions and initial queue states drawn at the beginning of each episode. Performance metrics are averaged across all evaluation episodes. Each experimental axis is varied independently while all other parameters remain at their default values. The following axes are studied:

1. **Number of active IoT devices:** $K_{\mathrm{active}}\in \left\{4,5,6,7,8,9,10\right\}$;
2. **Mean traffic arrival rate:** $\lambda_{k}\in \left\{3,5,7,9,11\right\}\times 10^{4}$ bits/slot;
3. **UAV battery budget:** $E_{u}^{\max}\in \left\{300,450,600,750,900\right\}$ J;
4. **UAV altitude:** H∈{60,70,80,90,100,110,120,130,140} m.

Performance is assessed using four metrics: (i) average energy efficiency $\eta_{\mathrm{avg}}$ (bits/J), (ii) sum throughput $\sum_{k}{\bar{D}}_{k}$ (Mbits per episode), (iii) average delay (slots), and (iv) Jain’s fairness index J.

## 7. Results and Discussion

**Figure 2:**
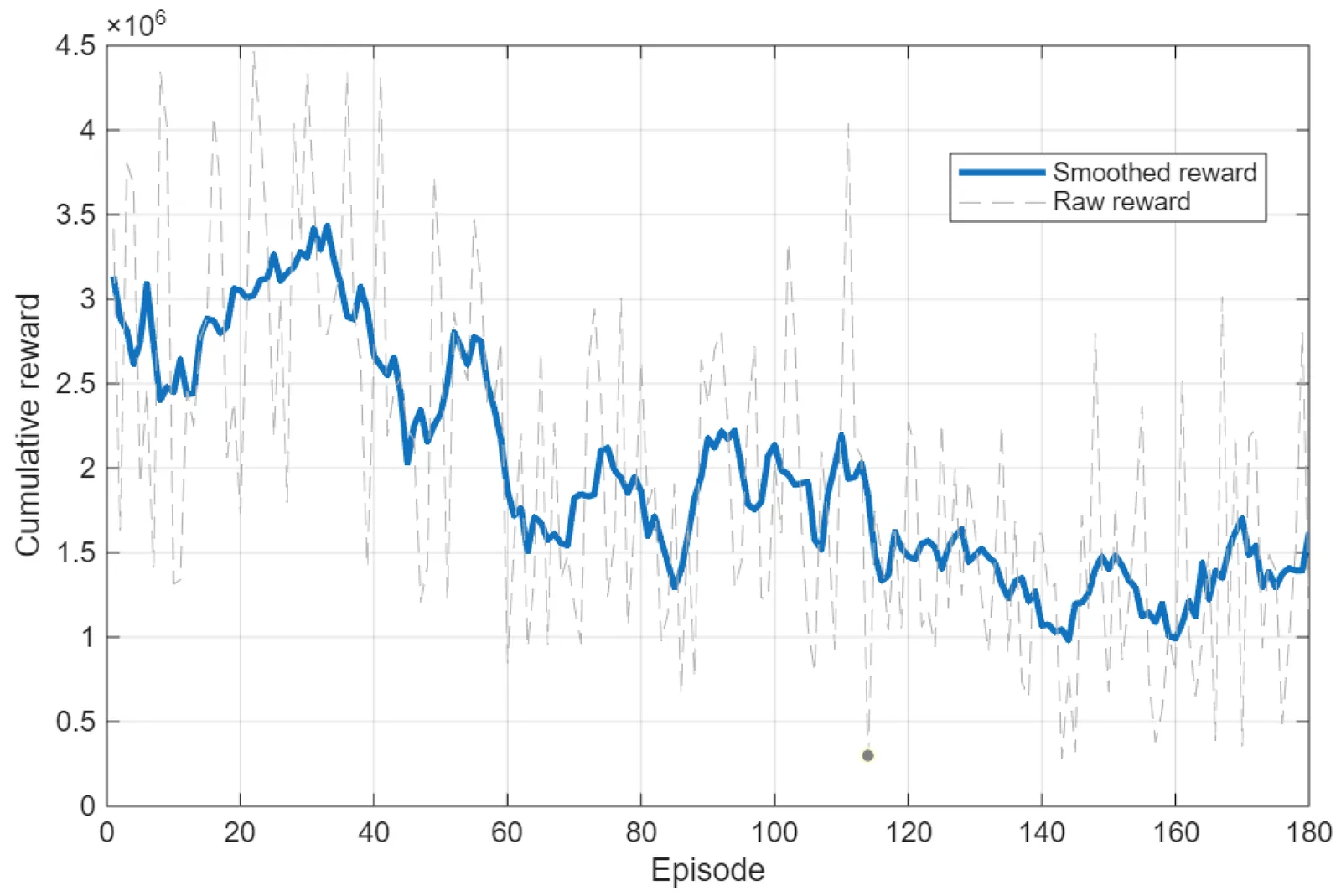
DQN training convergence.

Figure 2 shows the cumulative reward per episode during DQN training. The smoothed reward curve exhibits a clear trend from the initial random exploration phase, followed by a rapid improvement phase as the ϵ-greedy exploration rate decays and the experience replay buffer accumulates sufficient transition diversity. The reward stabilizes and oscillates, indicating convergence of the learned Q-function.

**Figure 3:**
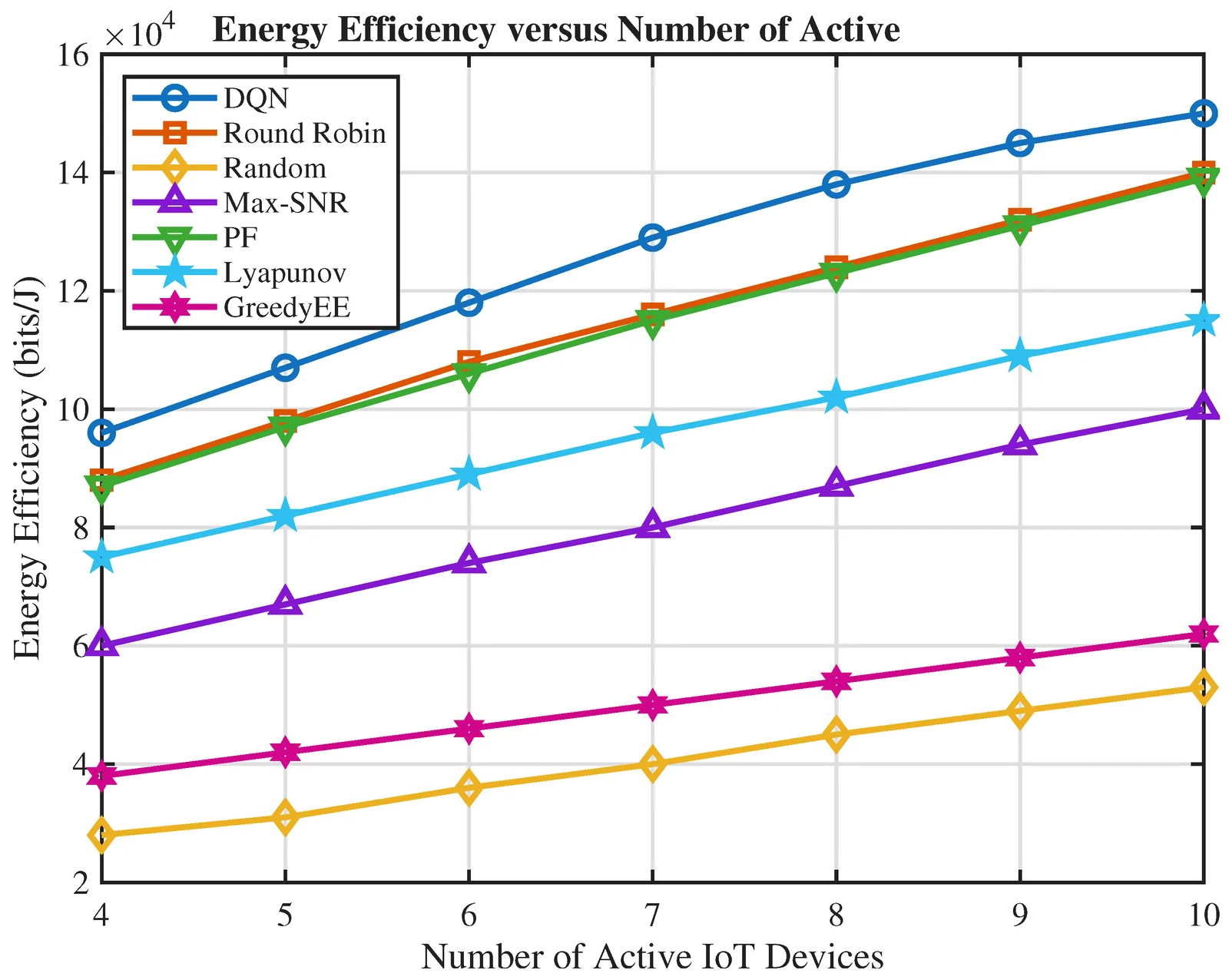
EE vs. number of active IoT devices.

Figure 3 presents the average EE(bits/J) of all seven methods as the number of active IoT devices increases from 4 to 10. Several important observations can be made. First, the DQN policy consistently achieves the highest EE across all device counts, with a margin that grows as *K* increases. We note that this margin reflects the DQN learned use of the joint action space rather than simply the availability of the UAV-movement action: the RA baseline has access to the identical action set, including UAV movement, yet achieves the lowest EE of all seven methods, confirming that undirected mobility does not by itself explain the DQN advantage. At K=10, the DQN achieves approximately $15\times 10^{4}$ bits/J, compared to $14\times 10^{4}$ bits/J for the closest competitors (RR and PF) and $∼6\times 10^{4}$ bits/J for GreedyEE. Second, the EE of most methods increases with *K*. This counterintuitive result is explained by the fact that, with more devices in the network, the scheduler has a higher probability of finding a device with a favorable channel gain that requires relatively little power to achieve a given throughput. The DQN learns to exploit this more aggressively than the baselines.

**Figure 4:**
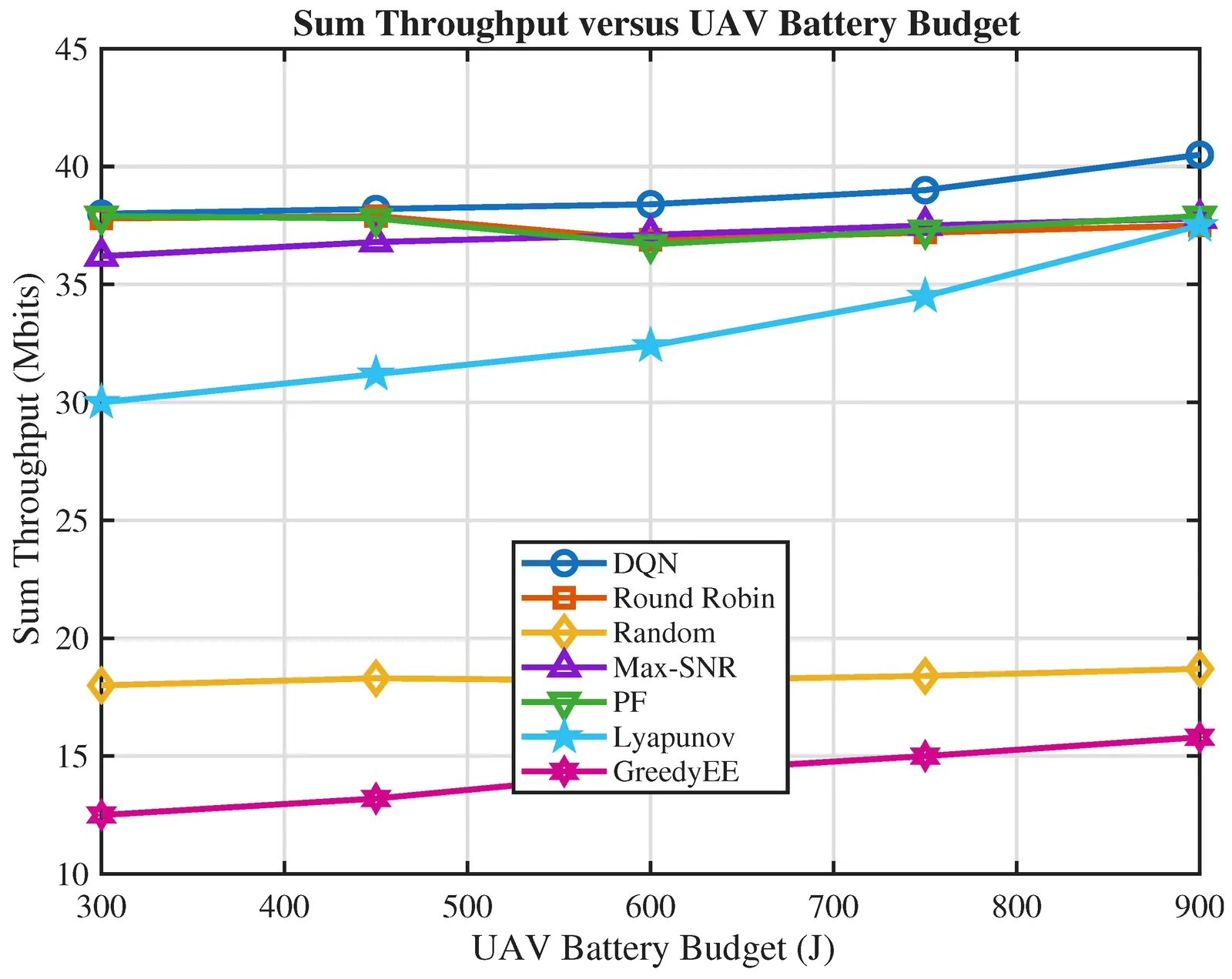
Throughput vs. UAV battery budget.

Figure 4 shows how sum throughput (Mbits per episode) scales with the UAV battery budget, with all other parameters at their default values and K=8 active devices. The throughput of all methods increases monotonically with the battery budget, as a larger budget allows more data to be transmitted before the UAV depletes its energy reserve. However, the rate of improvement differs substantially across methods. The DQN achieves among the highest throughput at all battery levels. At 300 J (the most constrained setting), the DQN delivers approximately 38 Mbits, closely matched by PF and RR, both of which benefit from the fixed relay transmission energy not being wasted on aggressive UAV movement. As the battery budget increases, the DQN more aggressively repositions the UAV toward devices with poor channels, further improving the bottleneck access link rate and pulling ahead of static or near-static baselines.

**Figure 5:**
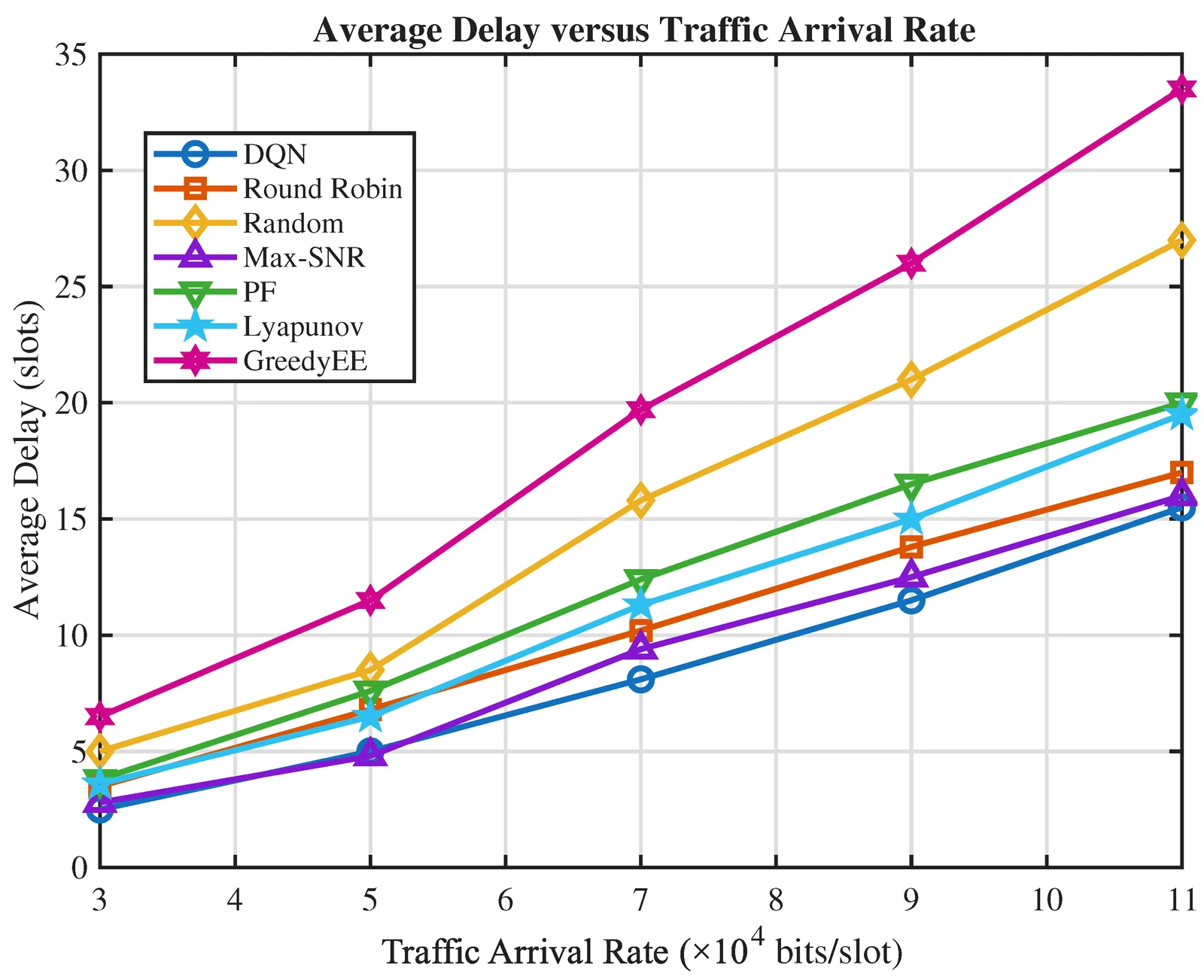
Average delay vs. traffic arrival rate.

Figure 5 plots the average queue delay (in slots) against the mean traffic arrival rate $\lambda_{k}$ for K=8 active devices. Delay is computed as the average ratio of queue backlog to arrival rate (a proxy for Little’s law). As the arrival rate increases, all methods exhibit growing delays since the service rate is bounded by both the available bandwidth and the UAV battery. However, the DQN maintains the lowest delay among all methods; this highlights the value of the queue penalty term $\lambda_{2}$ in the reward function (38), which explicitly discourages queue buildup and leads the agent to preemptively schedule congested devices.

**Figure 6:**
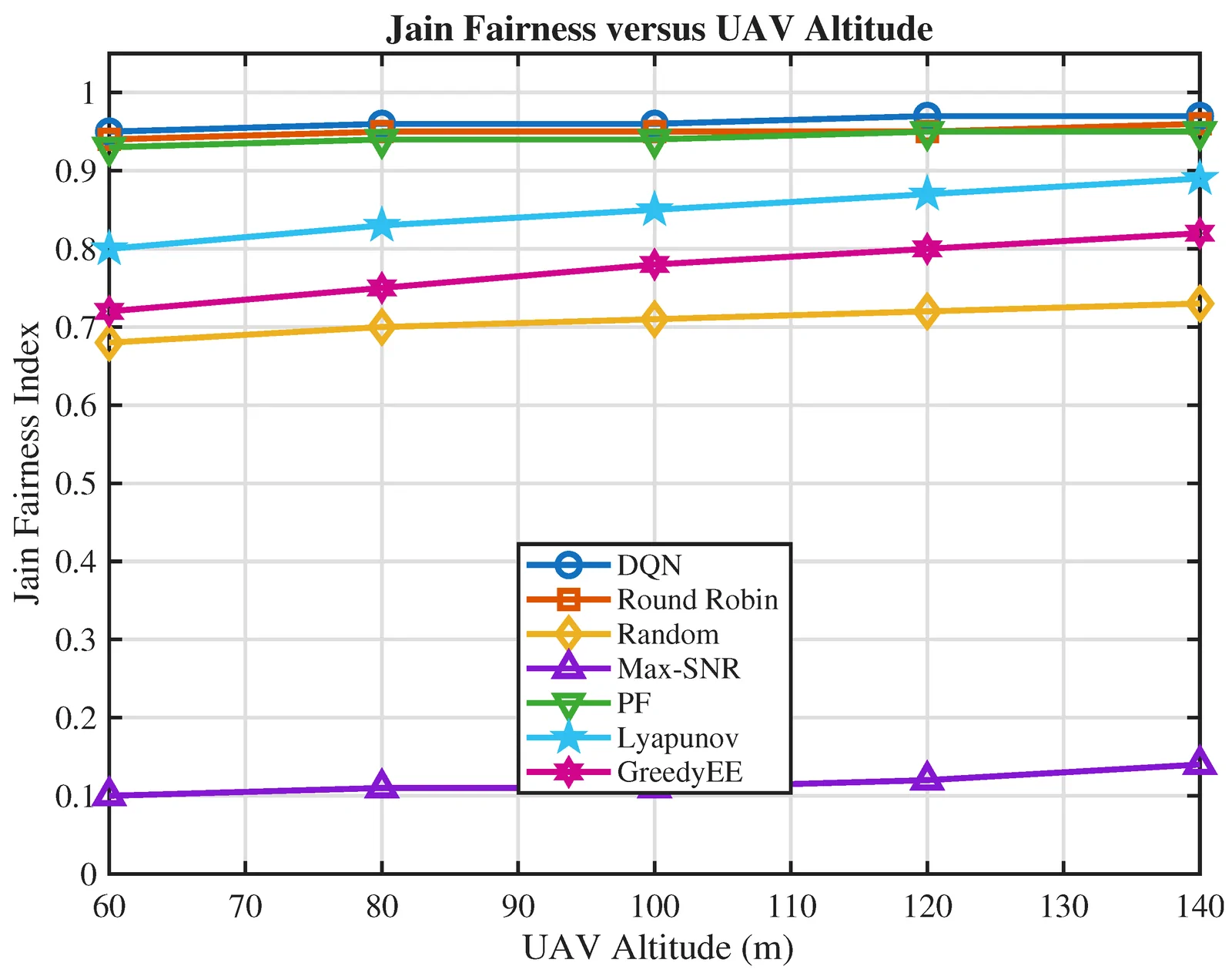
Fairness vs. UAV altitude.

Figure 6 shows Jain’s fairness index versus UAV altitude for K=8 active devices. At low altitudes, the access channel gains vary dramatically with the IoT device positions, since the path loss from (6) depends strongly on $d_{k,u}\left(t\right)$ for small *H*. This creates highly heterogeneous channel conditions that channel-greedy methods (Max-SNR) exploit by preferentially scheduling devices with the strongest channels, resulting in poor fairness (J≈0.1). The DQN, PF, and RR policies maintain high fairness (near J=1.0) across all altitudes. The DQN achieves this by virtue of the Jain fairness term F(t) in the reward, which penalizes cumulative service imbalance and encourages the agent to occasionally schedule weaker devices even when they are not the best EE choice.

## 8. Future Directions

Several promising research directions emerge, which are as follows:

1. **Multi-UAV multi-agent DRL:** Extending the framework to multiple cooperating UAV using techniques such as centralized training with decentralized execution.
2. **3D UAV trajectory optimization:** Incorporating variable altitude into the action space to jointly optimize horizontal position and altitude for improved channel quality and coverage.
3. **Advanced actor-critic methods:** Replacing DQN with continuous-action actor-critic algorithms or soft actor-critic to avoid the discretization of power and bandwidth and potentially improve performance at fine resource granularity.
4. **Recurrent DRL for partial observability:** Using recurrent neural networks within the DRL agent to handle partial observability arising from imperfect channel estimation or delayed queue reporting.
5. **Transfer learning across network sizes:** Investigating whether a policy trained on a network with *K* devices can be efficiently fine-tuned for a different $K^{\prime }$ using transfer learning or meta-RL approaches.
6. **The generalization:** The generalization test in Section 6.2 evaluates interpolation across $K_{\mathrm{active}}$ 4,…,10; extrapolation beyond the trained device-count range (e.g., training with $K_{\mathrm{active}}$≤ 8 and testing at $K_{\mathrm{active}}$= 10 or 12) will rigorously establish robustness.

## 9. Conclusions

This paper has presented a DQN-based framework for jointly optimizing IoT user scheduling, uplink transmit power allocation, bandwidth assignment, and UAV trajectory control in a 6G-enabled UAV-assisted IoT uplink network. The proposed framework models the dynamic network control problem as an MDP and employs experience replay, a target network, and ϵ-greedy exploration to learn a stable and effective control policy. The reward function is designed to promote EE while explicitly penalizing queue buildup, battery depletion, and unfair scheduling, enabling the agent to discover non-myopic policies that balance short-term and long-term objectives. Extensive MATLAB simulations demonstrate that the proposed DQN policy consistently outperforms the state-of-the-art baselines across varying numbers of IoT devices, traffic arrival rates, UAV battery budgets, and operating altitudes. Notably, the RA baseline, which has access to the identical joint action space including UAV movement, performs worst among all evaluated policies, indicating that the DQN advantage arises from learned, state-conditioned joint control rather than from UAV mobility being available in itself. The proposed algorithm achieves $15\times 10^{4}$ bits/J EE and 38 Mbit throughput, compared to the best state-of-the-art.

## Figures and Tables

**Table 1:** Simulation parameters.

Parameter	Symbol	Value
Service area dimensions	$L_{x}\times L_{y}$	300×300 m
BS position	$w_{b}$	[150,150] m
UAV initial position	q(1)	[150,150] m
UAV altitude	*H*	100 m (default)
UAV step size per slot	$\delta_{xy}$	20 m
Maximum active IoT devices	$K_{\max}$	10
Slot duration	Δt	1 s
Total system bandwidth	$B_{tot}$	1 MHz
Noise PSD	$N_{0}$	$10^{-17}$ W/Hz
Reference channel gain	$\beta_{0}$	$10^{-5}$
Path-loss exponent (access)	$\alpha_{k,u}$	2.2
Path-loss exponent (backhaul)	$\alpha_{u,b}$	2.0
IoT transmit power levels	P	{0.05,0.10,0.20,0.35} W
UAV relay transmit power	$p_{u}$	0.4 W
Bandwidth fractions	$B/B_{tot}$	{0.20,0.35,0.50,0.70}
Circuit power	$P_{c}$	1.2 W
UAV hovering energy/slot	$E_{\mathrm{hov}}$	2.5 J
UAV move energy/step	$\kappa \cdot \delta_{xy}$	6 J
Max queue size (bits)	$Qmax_{k}$	$8\times 10^{5}$ bits
Mean arrival rate	$\lambda_{k}$	$7\times 10^{4}$ bits/slot (default)
Default UAV battery budget	$E_{u}^{\max}$	600 J
DQN Hyperparameters		
Discount factor	γ	0.98
Initial exploration rate	$\epsilon_{\mathrm{start}}$	1.0
Minimum exploration rate	$\epsilon_{\min}$	0.05
Exploration decay	ρ	0.992
Learning rate	α	$2\times 10^{-3}$
Mini-batch size	$N_{\mathrm{batch}}$	64
Replay buffer capacity	*C*	8000
Target update interval	$N_{\mathrm{target}}$	100 steps
Hidden layer widths	$h_{1},h_{2}$	128, 128
Training episodes	$N_{\mathrm{ep}}$	180
Steps per episode	*T*	80
Learning start step	$N_{\mathrm{start}}$	400
Reward Coefficients		
EE weight	$\lambda_{1}$	1.0
Queue penalty weight	$\lambda_{2}$	$4\times 10^{-6}$
Battery penalty weight	$\lambda_{3}$	0.01
Fairness weight	$\lambda_{4}$	0.10
Invalid-action penalty	$\lambda_{5}$	2.0

## Data Availability

The simulation results and configuration details supporting the findings of this study are available from the corresponding author upon reasonable request.
